# Development and validation of an SNP genotyping array and construction of a high-density linkage map in castor

**DOI:** 10.1038/s41598-019-39967-9

**Published:** 2019-02-28

**Authors:** S. Senthilvel, Arpita Ghosh, Mobeen Shaik, Ranjan K. Shaw, Prashanth G. Bagali

**Affiliations:** 1 ICAR-Indian Institute of Oilseeds Research, Rajendranagar, Hyderabad, 500030 India; 2 Xcelris Labs Ltd., Xcellon building, Navrangpura, Ahmedabad, 380009 India

## Abstract

Castor is a commercially important oilseed crop that provides raw materials for several industries. Currently, the availability of genomic resources for castor is very limited. In this study, genome-wide SNPs were discovered in castor via whole-genome sequencing of 14 diverse lines to an average of 34X coverage. A total of 2,179,759 putative SNPs were detected, and a genotyping array was designed with 6,000 high-quality SNPs representing 2,492 scaffolds of the draft castor genome (87.5% genome coverage). The array was validated by genotyping a panel of 314 inbred castor lines, which resulted in 5,025 scorable SNPs with a high call rate (98%) and reproducibility (100%). Using this array, a consensus linkage map consisting of 1,978 SNP loci was constructed with an average inter-marker distance of 0.55 cM. The genome-wide SNP data, the genotyping array and the dense linkage map are valuable genomic tools for promoting high-throughput genomic research and molecular breeding in castor.

## Introduction

Castor (*Ricinus communis* L.) is the only species within the genus, *Ricinus* in the Euphorbiaceae family, that is both an autogamous and allogamous species with a diploid number of chromosomes (2n = 2x = 20) and a genome of approximately 320 Mb in size^1^. It is an industrially important oilseed crop and the only source of ricinoleic acid (12-hydroxy-octadeca-9-enoic acid) in plants. Castor oil and its derivatives are used in the manufacturing lubricants, textiles, paper, plastics, paints, inks, cosmetics and pharmaceuticals. The demand for castor is also boosted by its potential for biofuel production owing to its wide adaptability, high productivity and high oil yield^2^. However, to increase castor production, elite high-yielding cultivars that display resistance to biotic and abiotic stresses need to be developed. Such cultivar development can be accelerated if molecular markers are integrated into current castor breeding programmes, as proven in other crops.

A lack of genomic resources and the absence of a high-throughput genotyping system have impeded marker-assisted breeding in castor. Currently, a draft genome assembly in the form of 25,800 scaffolds^3^ and a few thousand EST sequences are the only publicly available resources. A linkage map with 331 markers, mostly SSR, has been constructed with an average inter-marker distance of 3.63 cM and many gaps of more than 10 cM^4^. Of the few studies in castor on marker-trait associations, there were attempts to identify RAPD markers linked to Fusarium wilt resistance using bulked segregant analysis in an F_2_ population^5,6^. Recently, QTLs for resistance to charcoal rot have been identified using a mapping population of F_2:3_ families^7^.

At present, markers based on single nucleotide polymorphisms (SNPs) hold promise in genetic studies and breeding applications and can assist in linkage map construction, marker-trait association, and marker-assisted selection because of their abundance, high level of polymorphism, high throughput capability, and cost-effectiveness. SNP markers could be the preferred marker system for crop species, especially oilseeds, in which general microsatellite diversity appears to be low^8–10^. Moreover, a low level of SSR allelic diversity has been reported in castor^11,12^. In a previous study, Foster and colleagues^13^ used 48 SNP loci to assess genetic diversity in castor and underpinned the need for a large number of marker loci for low-diversity species like castor.

With the advent of next-generation sequencing (NGS) technologies, generating genome-wide markers is no longer a limitation for any species. NGS technologies have drastically increased the speed at which a genome sequence can be generated, while greatly reducing costs. Thus, the discovery of genome-wide SNPs has become very simple and inexpensive^14^. Concurrently, several high-throughput SNP array-based genotyping platforms have been developed^15^. Recently, Genotyping by Sequencing (GBS) has become a popular high-throughput marker detection platform^16^. GBS is an NGS protocol employed to simultaneously discover and score segregating markers in populations of interest by sequencing highly multiplexed reduced representation libraries of samples^17^. However, at present, this technique has certain limitations for crop genotyping. A major complication is the high-level DNA sequence duplication in angiosperms. The critical level of sequencing read depth for a given experimental design can be affected by factors such as genome size, genome complexity, ploidy, expected heterozygosity, and others. Poor coverage of specific loci lead to an excessive number of missing values, compromising the accuracy of genetic studies^18^. Moreover, the experimental operation of GBS and data analysis are beyond the capabilities of an average breeding programme; in contrast, ‘genotyping arrays’ can be used to genotype many samples within a short period, and the data analysis is relatively easy^19^.

In this context, this study was undertaken with the following objectives: (i) to discover genome-wide SNPs in castor through whole-genome sequencing of a set of diverse genotypes, (ii) to develop and validate an SNP genotyping array, and (iii) to construct a dense linkage map for high-throughput genomic applications in castor.

## Results

### Whole-genome sequencing and discovery of SNPs

A set of 14 diverse castor genotypes, including cultivars and germplasm accessions, was chosen for whole-genome sequencing (Table 1). Paired-end (PE) sequencing libraries were generated using the genomic DNA of all samples. The libraries were of high quality per the sizing profiles in Agilent HS Chip. The fragment size of the libraries ranged from 402 bp to 875 bp. Sequencing of these libraries using Illumina’s MiSeq (3 genotypes) and HiSeq (11 genotypes) platforms yielded 5.4 to 22.0 Gb of equivalent data per sample with 15X to 62X coverage. In total, 169 Gb of DNA sequence (755 million 2 × 100 bp reads and 36 million 2 × 250 bp reads) data were generated for 14 castor genotypes. The number of reads per sample ranged from 11.3 to 108.9 million (Table 2). The huge difference in the number of reads among samples was due to the use of two different sequencing platforms (MiSeq and HiSeq by Illumina). The MiSeq platform yielded an average of 12.2 million reads (6.2 Gb data), whereas HiSeq yielded 68.7 million reads (13.7 Gb data).

**Table 1:** Characteristics of castor inbred lines used for sequencing.

Genotype	Morphological features	Special attribute
JI220	Medium height, green stem, triple bloom, spiny capsule, normal internode, flat leaves, monoecious inflorescence	Elite inbred line, resistant to Fusarium wilt
JC12	Medium height, red stem, double bloom, spiny capsule, normal internode, flat leaves, monoecious inflorescence	Elite inbred line, parent of a mapping population, resistant to reniform nematode
TMV5	Medium height, red stem, triple bloom, spiny capsule, normal internode, flat leaves, monoecious inflorescence	Commercial variety
RG43	Dwarf, red stem, triple bloom, spiny capsule, normal internode, flat leaves, monoecious inflorescence	Germplasm accession, resistant to Fusarium wilt, reniform nematode and leafhopper (*Empoasca flavescens*)
RG72	Dwarf, red stem, double bloom, spiny capsule, normal internode, flat leaves, monoecious inflorescence	Germplasm accession, early maturing and drought tolerant
RG3309	Tall, Magogany stem, double bloom, short-spiny capsule, normal internode, flat leaves, monoecious inflorescence	Germplasm accession, moderately resistant to gray mold and resistant to Fusarium wilt
DPC9	Medium height, green stem, no bloom, spiny capsule, normal internode, flat leaves, pistillate inflorescence	Female parent of a commercial hybrid
48-1	Medium height, red stem, doube bloom, non-spiny capsule, normal internode, flat leaves, monoecious inflorescence	Commercial variety, parent of a mapping population, resistant to Fusarium wilt
RG1139	Medium height, red stem, no bloom, short-spiny capsule, normal internode, flat leaves, monoecious inflorescence	Germplasm accession, parent of a mapping population, moderately resistant to gray mold
RG2787	Tall, Mahogany stem, double bloom, spiny capsule, normal internode, flat leaves, monoecious inflorescence	Germplasm accession, resistant to Macrophomina root rot and Fusarium wilt
RG2819	Tall, green stem, double bloom, spiny capsule, normal internode, flat leaves, monoecious inflorescence	Germplasm accession, resistant to Macrophomina root rot and Fusarium wilt
VP1	Dwarf, green stem, triple bloom, spiny capsule, condensed internode, cup shaped leaves, pistillate inflorescence	Female parent of a commercial hybrid
DCS9	Medium height, red stem, double bloom, spiny capsule, normal internode, flat leaves, monoecious inflorescence	Commercial variety and parental line of a mapping population
RG3216	Medium height, red stem, no bloom, short-spiny capsule, normal internode, flat leaves, monoecious inflorescence	Germplasm accession, parent of a mapping population

**Table 2:** Statistics on whole genome sequencing of 14 castor inbred lines.

Genotype	Number of raw reads	Total data (Gb)	Number of filtered reads	Alignment (%)	Genome coverage (%)	Platform used
JI220	11,251,618	5.40	10,531,223	89.76	94.63	MiSeq
JC12	12,626,709	7.20	11,906,343	96.91	94.97	MiSeq
TMV5	62,392,969	12.47	57,398,835	97.36	95.57	HiSeq
RG72	59,087,998	11.80	55,979,084	96.51	95.29	HiSeq
RG3309	70,686,223	14.13	66,770,385	95.66	95.04	HiSeq
DPC9	65,820,623	13.10	60,207,321	96.37	95.50	HiSeq
RG43	12,787,405	5.90	11,622,830	87.41	92.84	MiSeq
48-1	51,270,973	10.25	48,407,365	97.26	95.50	HiSeq
RG1139	73,466,140	14.69	69,352,963	97.83	97.02	HiSeq
RG2787	63,835,573	12.76	58,743,049	95.58	95.57	HiSeq
RG2819	62,782,508	12.55	58,926,757	96.90	95.70	HiSeq
VP1	62,126,053	12.42	58,885,533	98.09	95.63	HiSeq
DCS9	75,062,342	15.00	62,244,984	97.41	94.87	HiSeq
RG3216	108,880,111	22.00	91,131,064	97.73	97.39	HiSeq

The high-quality filtered reads were aligned with the reference genome. The mapping percentage ranged from 87.4 to 98.1. On an average, 95.8 per cent of the reads could be mapped onto the reference genome. A good level of genome coverage was observed. The per cent genome coverage (without Ns) ranged from 92.8 to 97.4, with an average of 95.4. The number of SNPs/InDels identified between sample and reference ranged from 294,956 to 929,114 (Table 3). A total of 2,179,759 SNPs were identified across the samples, with a frequency of one SNP per 160 bp.

**Table 3:** Statistics on SNPs/InDels identified between the samples and the reference.

Genotype	Total SNPs identified	Transition	Transversions	Triallelic SNPs	Number of SNPs after filtration	Genic SNPs
JI220	294,956	167,623	78,092	51	55,587	29,439
JC12	929,114	577,558	245,896	24	63,221	84,792
TMV5	713,512	453,056	192,247	9	74,178	73,923
RG72	644,037	406,047	172,030	5	82,802	62,838
RG3309	852,482	225,033	538,652	6	105,300	80,195
DPC9	604,063	380,789	161,192	6	78,457	56,241
RG43	387,296	235,152	100,703	27	62,842	32,010
48-1	592,812	373,094	160,160	4	78,747	61,035
RG1139	490,382	295,749	129,786	2	74,252	63,802
RG2787	670,889	423,464	176,940	11	88,115	60,934
RG2819	672,843	425,258	178,583	10	88,806	61,609
VP1	440,832	266,069	118,137	2	73,224	53,660
DCS9	305,433	187,765	90,138	0	111,179	39,500
RG3216	415,777	117,476	56,680	0	74,089	63,916

### Genotyping array design

The putative SNPs identified through sequencing of 14 castor genotypes were further filtered for inclusion in genotyping assay development. On on average, 15 per cent of SNPs remained after filtering out the (i) SNPs with a less than 75 bp flanking region, (ii) SNPs located within 100 bp from the target SNP, (iii) insertion or deletion variants (InDels), and (iv) heterozygous SNPs. After filtration, the total number of common SNPs across 14 samples was 130,666, of which 21,185 were from genic regions. From this set, 10,872 SNPs were initially selected for array design, considering their functional roles (fatty acid biosynthesis, oil metabolism, disease resistance, and ricin biosynthesis) and genomic coverage (maximum possible representations of scaffolds of the reference genome). Out of 10,872 SNPs, we finally selected 6,000 SNPs, that required only one bead type for genotyping and had an assay design score of >0.7 for assay development. The details of the SNPs selected for array design are given in Supplementary Table S1. The set of SNPs in the array represented 2,492 scaffolds of draft castor genome, with genome coverage of 87.5 per cent. Out of 6,000 SNPs in the final array, 3,619 SNPs were from genic regions.

### Validation of genotyping array

The technical performance and the practical utility of the SNP array were assessed by genotyping a panel of 314 inbred castor lines. Of the 6,000 targeted SNPs, only 5,238 could be included on the Infinium BeadChips. Out of the 5,238 SNPs present on the Infinium BeadChips, high-quality genotype calls were obtained for 5,038 SNPs, while clear genotype clustering was not obtained for the remaining 200 SNPs.

**Figure 1:**
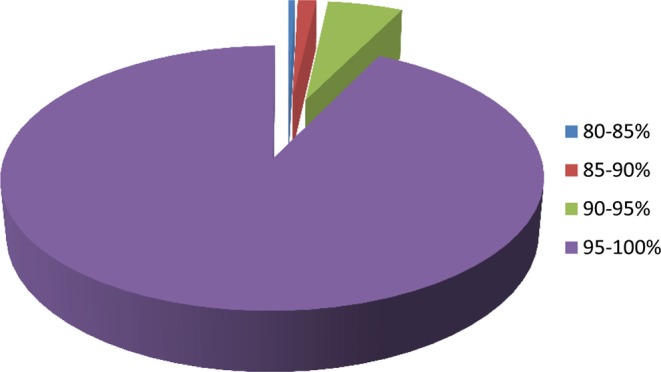
Distribution of call rate of SNPs across 314 castor inbred lines.

Out of 5,038 scorable SNPs, 927 were monomorphic (19%) and 13 SNPs had missing data points of more than 20 per cent in the panel of 314 lines. Finally, 4,098 SNPs were called successfully with high confidence. The GeneTrain and GeneCallC50 scores of all the SNPs were >0.4. The call rate of SNPs ranged from 80 to 100 per cent, with an average of 98 per cent (Fig. 1). The reproducibility of all 4,098 SNPs across technical and biological replicates was 100 and 99.62 per cent, respectively. From these parameters, it is very evident that the genotyping quality and reliability of this array are very high.

**Figure 2:**
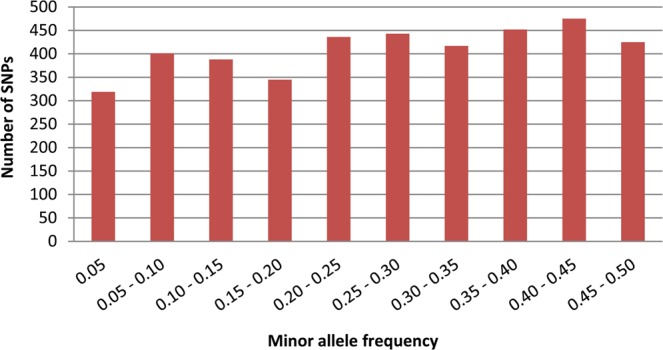
Distribution of minor allele frequency (MAF) of SNPs in the array.

The biological utility of the SNP array was assessed by analyzing the genetic diversity and relationships in the genotype panel. Of the 4,098 informative SNPs, 2,690 (65.64%) had an MAF of >0.2 and could be considered as markers with normal allele frequencies. Approximately 18 per cent of SNPs had an MAF of 0.1 to 0.2. A total of 291 SNPs had an MAF of <0.05. In addition, 489 SNPs (12%) showed almost equal allele frequencies (with MAF close to 0.5) for two alternative alleles. The distribution of MAF is shown in Fig. 2.

**Figure 3:**
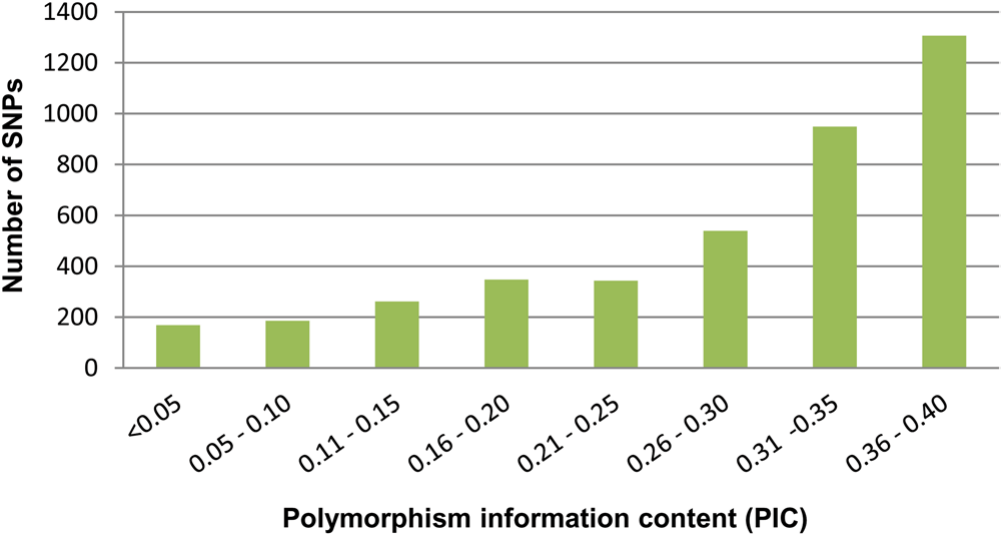
Distribution of polymorphism information content (PIC) values of SNPs in the array.

The polymorphism information content (PIC) values for individual SNPs ranged from 0.003 to 0.375, with the peak distribution at >0.3 (Fig. 3). The average observed heterozygosity across the castor lines was 0.11, and the expected heterozygosity (gene diversity) ranged from 0.003 to 0.500, with an average of 0.351. A neighbour-joining tree was constructed using the genotypic data from 4,098 SNP loci in 314 castor lines. The genetic relationship revealed by the dendrogram was as expected based on the pedigree and breeding history. For instance, a pistillate line, DPC9, and its nine mutant selections were placed together. Similarly, the inbred lines with shared pedigrees were in close proximity in the dendrogram (Supplementary Fig. 1).

### Construction of linkage map

#### Linkage map of JC12×48-1 cross

The RIL population of the JC12 × 48-1 cross, along with their parents, were genotyped using the SNP array. A total of 1,090 SNP loci were polymorphic and segregated in the population, of which 246 loci (22%) showed segregation distortion towards either of their parents. Out of 1,090 SNPs, 1,028 showed unique segregation. Linkage analysis was performed using the genotypic data from all 1,090 SNP markers. All markers could be mapped, and a genetic linkage map was constructed with 10 linkage groups, corresponding to the number of haploid chromosomes. The total length of the map was 1,139.8 cM. The number of markers per linkage group ranged from 82 to 207 (Table 4). The map was very dense, with an average inter-marker distance of 1.12 cM. The longest gap was 12.4 cM. Only seven marker intervals were longer than 10 cM. The map is provided in Supplementary Table S2.

**Table 4:** Details of linkage maps constructed.

Linkage group	(JC12 × 48-1 map)			(DCS9 × RG1139 map)			Consensus map		
	No. of markers	Map length (cM)	Average distance between markers (cM)	No. of markers	Map length (cM)	Average distance between markers (cM)	No. of markers	Map length (cM)	Average distance between markers (cM)
LG1	207	200.0	1.07	182	148.2	0.91	314	155.1	0.54
LG2	125	92.1	0.76	132	60.8	0.59	228	83.8	0.43
LG3	107	105.6	1.08	155	102.9	0.75	207	105.6	0.54
LG4	105	139.4	1.38	74	73.0	1.12	147	75.4	0.55
LG5	83	128.2	1.62	74	84.5	1.28	135	86.9	0.70
LG6	74	96.5	1.38	85	93.4	1.18	137	96.8	0.74
LG7	139	105.6	0.80	174	105.9	0.68	249	105.9	0.45
LG8	82	89.1	1.14	169	74.1	0.48	215	84.9	0.43
LG9	102	95.5	1.06	183	115.1	0.73	249	115.1	0.53
LG10	66	87.8	1.42	45	46.9	1.20	103	86.4	0.92
Total map	1090	1139.8	1.12	1273	904.8	0.81	1978	995.8	0.55

#### Linkage map of DCS9×RG1139 cross

The RILs of the DCS9 × RG1139 cross, along with their parents, were genotyped using the SNP array. A total of 1,273 SNP loci were scored as polymorphic in the population. About 28 per cent of the loci showed skewed segregation. Linkage analysis was performed using the genotypic data from all SNP markers, and a genetic linkage map was constructed with 10 linkage groups. The total length of the map was 904.8 cM. The number of markers per linkage group ranged from 45 to 183 (Table 4). The map was very dense, with average inter-marker distance of 0.81 cM. The longest gap was 8.0 cM. The map is provided in Supplementary Table S3.

#### Consensus map

**Figure 4:**
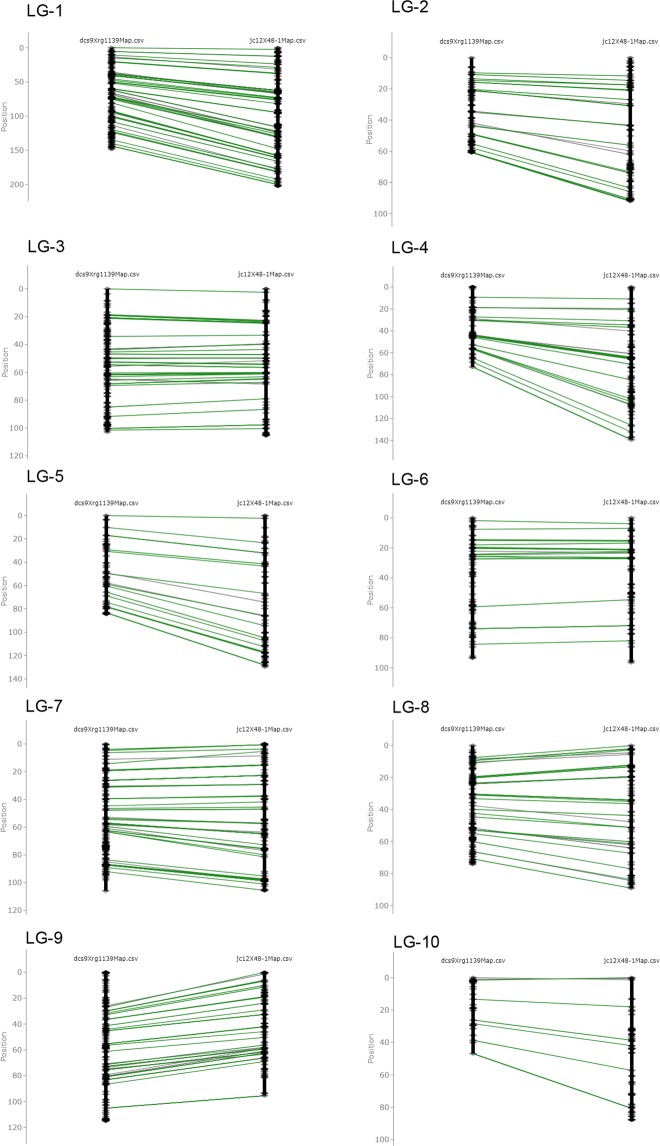
Comparison of DCS9 × RG1139 and JC12 × 48-1 maps.

There were 392 markers in common between the two crosses, which enabled the construction of a consensus map. The number of common markers per linkage group ranged from 9 (LG-10) to 75 (LG-1). The consensus map was composed of 1,978 SNP markers and spanned a total length of 995.8 cM with an average distance of 0.55 cM between adjacent markers. The marker orders in the consensus map were in general consistent with the individual genetic linkage maps (Fig. 4). The map is provided in Supplementary Table S4.

The pairwise comparison of the linkage maps indicated no potential chromosomal rearrangements, and none of the markers were found to map to different LGs.

## Discussion

Even though castor is an industrially important crop, it has not yet benefited from the genomic revolution. The availability of genomic resources for castor is very limited. This study attempted to generate genomic resources, *viz*., genome-wide SNP loci, a genotyping array and a linkage map.

To identify genome-wide SNPs, whole-genome sequence data for 14 inbred castor lines were generated with very high coverage (average 34X) and mapped to the reference genome with genome coverage (without Ns) of 92.8 to 97.4 per cent. The high mapping percentage achieved in this study might be due to the use of paired-end sequencing, which helps to precisely map the short reads onto the reference. In sorghum, Nelson and his collegues^20^ could map only 30 per cent of the reads when they used single-end reads from restriction site associated DNA sequences. By aligning the sample reads with the reference, a total of 2,179,759 SNPs were identified across samples, with a frequency of one SNP per 160 bp. The SNP frequency observed in castor appears to be higher than that observed in self-pollinated crops such as pea (1 SNP/540 bp)^21^, soybean (1 SNP/490 bp)^22^, rice (1 SNP/238 bp)^23^, and wheat (1 SNP/233 bp)^23^ but lower than in cross-pollinated perennial species such as *Eucalyptus* (1 SNP/16 bp)^24^ and oil palm (1 SNP/74 bp)^25^. However, it is important to note that the frequencies of SNP occurrence reported in the above-mentioned studies might be over/under estimated because those studies were based on only a few genes or transcriptomes, not whole genomes. The frequency of SNP occurrence varies widely among crops depending upon their domestication history, their reproductive habits (autogamous or allogamous), the diversity of the populations under assessment, and the status (coding or non-coding) of the analysed regions^21^. From the large catalogue of SNPs, a genotyping array with 6,000 carefully chosen SNPs was designed. The set of SNPs in the array represented 2,492 scaffolds of the draft castor genome with genome coverage of 87.5 per cent. Out of 6,000 SNPs in the final array, 3,619 were from genic regions. SNPs in genic regions could potentially be more informative in genome-wide association studies.

To assess the utility of the SNP array, a set of 314 castor lines representing the whole spectrum of the diversity available in the species was genotyped using the SNP array. The genotype panel comprised accessions of core germplasm, trait-specific germplasm lines, elite breeding lines, commercial varieties, and parents of commercial hybrids. Of the 6,000 targeted SNPs, only 5,238 could be included on the Infinium BeadChips. The manufacturing loss (12.7%) was within the expected limits and comparable to that in other studies^26–29^. Out of 5,238 SNPs present on the Infinium BeadChips, high-quality genotype calls were obtained for 5,038 SNPs. The success rate observed in this study (96.2%) was higher than the typical ≥90% rate obtained earlier with other crops. The high success rate might be due to the high sequencing depth and stringent filtration criteria applied while selecting SNPs for assay development. We carefully removed additional polymorphisms in the flanking sequences of target SNPs. The lower success rates observed in other studies could be due to their lack of specific selection for conserved SNP flanking sequences during genotype assay development. For example, the success rate in conifers was 69 to 77 per cent^30^, and in *Pinus pinaster* was 61 to 73 per cent^31^, when no specific selection for more conserved SNP flanking sequences was carried out.

Around 19 per cent of the SNPs were scored as monomorphic. Such a high level of false positives may have resulted from the use of draft assembly for SNP prediction. It is expected that reads from regions missing in a draft reference may map incorrectly to other regions of the assembly and lead to false SNP predictions. These would then show as monomorphic in the Illumina assay. Nevertheless, the technical performance of the array was superior, as evidenced by the high average call rate (98%) and reproducibility (100%).

The diversity parameters derived from the genotypic data for 314 inbred castor lines representing the whole spectrum of the diversity available in the species indicated only a moderate level of genetic diversity in castor. The average observed heterozygosity was 0.11, which is within the expected range of residual heterozygosity reported earlier^13,32^. The expected heterozygosity (gene diversity) ranged from 0.003 to 0.500, with an average of 0.351. The low level of heterozygosity could be due to preferential selection of SNPs in genic regions, which usually show reduced diversity. Previous studies have also reported low to moderate gene diversity estimates irrespective of the marker system. Allan and his collegues^11^ surveyed 41 germplasm accessions from five continents and 35 countries using AFLPs (3 primer combinations) and SSRs (9 primer pairs) and found that the average gene diversity was 0.126 for AFLPs and 0.239 for SSRs. Qiu and his collegues^32^ reported a relatively higher level of gene diversity (*He* = 0.41) by genotyping 24 accessions representing the main germplasms of castor from 14 countries using 118 polymorphic EST-SSR markers. Comparable levels of gene diversity (*He* = 0.38) were observed in our earlier study, in which 144 inbred lines derived from the core germplasm collection of castor were genotyped using 45 SSR markers^12^. The gene diversity estimate is generally influenced by the number of marker loci and the number/representation of genotypes used in the study. As the present study involved a large number of genotypes (more than 300 lines comprising core germplasm, breeding lines, and cultivars) and markers (over 4,000 SNP loci), we can confidently infer that only a moderate level of molecular diversity prevails in castor, despite its being a predominantly outcrossing species. The low level of diversity in castor might be due to its monotypic species status.

The genetic linkage map is a critical tool for molecular genetic studies and plant breeding applications. It is an important tool for physical mapping of genomes. High-density linkage maps have direct applications in marker-assisted selection through tight linkage of markers with the gene of interest. High-density genetic linkage maps are also useful in orienting and anchoring scaffolds arising from the genome sequence data^33,34^. To date, only two linkage maps largely based on SSR markers have been constructed for castor^4,7^. In this study, two RIL populations were used to construct a linkage map. The SNP array was used to genotype the population. The total number of polymorphic markers was 1,090 in the JC12 × 48-1 cross and 1,978 in the DCS9 × RG1139 cross. The proportion of skewed markers was more or less similar in both populations (22% in JC12 × 48-1 and 28% in DCS9 × RG1139); however, only 40 markers were shared across populations, indicating that the segregation distortion is specific neither to the cross nor the genomic region. The maps are fairly dense. The average inter-marker distance is 1.12 cM in the JC12 × 48-1 map and 0.81 cM in the DCS9 × RG1139 map.

It is interesting to note that the maps differ in total length, even though both were derived from RIL populations. The DCS9 × RG1139 map (904.8 cM) was smaller than the JC12 × 48-1 map (1,139.8 cM), which may be attributed to reduced recombination in DCS9 × RG1139^35^. In general, the maps of inter-specific crosses are significantly shorter than those of intra-specific crosses^36^. This was not the case in the present study, although RG1139 is a wild collection, whereas the other parents are improved breeding lines. Even though the individual populations differed in the extent of recombination, the marker order between individual maps was largely conserved (Fig. 4).

A consensus map was constructed using 392 markers that were shared between the two maps. The consensus map covered 995.8 cM and included 1,978 SNP markers, with only one gap of >10 cM and nine other gaps of >5 cM. The length of the consensus map was in between those of the individual genetic maps. A total of 722 scaffolds of the draft castor genome, covering 185.13 MB were anchored onto the consensus map. These anchored genome sequences could be used as a source for the development of additional markers^37^ and provide a valuable tool for fine-mapping and map-based cloning.

In summary, the SNP array developed in this study provides a valuable tool for high-throughput and cost-effective genotyping and mapping applications in castor. The genotyping array will provide the required resolution for discovery of marker-trait association through linkage and association analysis, evaluation of genetic variations, unravelling the genetic architecture of key quantitative traits, and exercising genomic selection in castor. The high-density linkage map can act as a reference for genetic and molecular studies in castor. The map can be readily used for (i) improving the genomic assembly of castor, (ii) QTL identification for several important traits, (iii) reference in constructing framework maps for individual populations, (iv) comparative genetic and genomic studies with related crops like cassava, and (v) identification of novel genes/alleles from related species.

## Materials and Methods

### Materials

#### Candidate genotypes for sequencing

A set of 14 inbred castor lines was chosen for whole genome-sequencing based on their genetic distinctness and utility in genetic studies and breeding programmes. The genotype panel represented wild (germplasm accessions) and cultivated (commercial varieties, parental lines of commercial hybrids, and parents of mapping populations) genepools. The details of the selected lines are given in Table 1.

### Genotype panel for validation of SNP array

The SNP array designed in this study was validated by genotyping a set of 314 inbred castor lines. The composition of the genotype panel included (i) a castor core germplasm set derived from a global collection of over 3,000 accessions, (ii) known sources for important agronomic traits, (iii) elite inbred lines developed from major castor breeding centres in India, and (iv) varieties and parental lines of hybrids released for cultivation; thus, the panel fairly represented the diversity available in castor.

### Mapping populations

Two recombinant inbred line (RIL) populations derived from the crosses JC12 × 48-1 and DCS9 × RG1139 were used to construct of linkage maps. The parents differed on range agronomic traits, including resistance to gray mold disease caused by *Botryotinia ricini*, vascular wilt caused by *Fusarium oxysporum* f.sp. *ricini*, and reniform nematode (*Rotylenchulus reniformis*). One true F_1_ plant of both the crosses was advanced to F_2_ by selfing. The individual F_2_ plants were further advanced up to F_6_ generation through the single-seed descent method. Finally, a total of 185 F_6_-RILs of JC12 × 48-1 and 188 F_6_-RILs of DCS9 × RG1139 were used for linkage map construction.

## Methods

### Whole-genome sequencing

Genomic DNA from 14 candidate genotypes was isolated using the CTAB method. The quality of the DNA was checked through 0.8% agarose gel electrophoresis and quantified using a Qubit® Fluorometer. Paired-end (PE) whole-genome sequencing (WGS) libraries were generated using the sample preparation kit from Illumina (Illumina Inc., USA) per the manufacturer’s protocol. Fragmentation of genomic DNA (1 µg) was carried out using the Covaris S2 System (Covaris, Inc., USA) followed by adapter ligation. Enrichment of adapter-modified DNA fragments by PCR was performed, and the enriched libraries were analyzed for size and quality using a high-sensitivity DNA kit on the Bioanalyzer (Agilent Technologies, USA). A paired-end cluster generation kit (Illumina Inc., USA) was used for cluster generation on paired-end flow cells. Cluster generation was carried out by hybridization of WGS libraries with a concentration ranging from 9 to 55 nmol/L, which was further diluted to make a stock solution of 4 nmol of molecules on the oligonucleotide-coated surface of the flow cell. Isothermal amplification of the libraries was carried out to generate clonal DNA clusters. Eleven libraries were multiplexed for sequencing on five different lanes of HiSeq2000 (Illumina Inc., USA) with an average read length of 2 × 100 bp. Three libraries were individually sequenced on MiSeq (Illumina Inc., USA) with an average read length of 2 × 250 bp.

### Discovery of genome-wide SNPs

The raw reads were filtered using Trimmomatic v0.17^38^ using the following criteria: (i) Slidingwindow: Perform a sliding window trimming of 25 bp, cutting once the average quality within the window falls below a threshold of 25; (ii) Leading: Cut bases off the start of a read, if below a threshold quality of 25; (iii) Trailing: Cut bases off the end of a read, if below a threshold quality of 25; (iv) Minlength: Drop the read if it is below 50 bp in length; and (v) Sequencing adapter trimming.

An available draft genome sequence of *Ricinus communis* L. with a 350 Mb genome size^3^ was downloaded from http://castorbean.jcvi.org/downloads.php. The filtered sample reads were aligned with the reference genome using BWA software^39^ with default parameters. SNP discovery and filtration were carried out using SAMtools v0.1.19 (mpileup and varFilter)^40^ with default parameters. The SNPs were filtered based on the following parameters: (i) minimum RMS mapping quality for SNPs: 25; (ii) minimum read depth: 8; (iii) window size for occurrences of one SNP: 100 bp; and (iv) window size for filtering dense SNPs: 1.

### Design of genotyping array

A genotyping array was developed on the Illumina Infinium platform (Illumina Inc., USA) with 6,000 carefully chosen SNPs. From the set of SNPs that passed the technical quality filters, as described in section 2.3, further filtration was carried out by removing (i) the SNPs with minor allele frequency of <0.2, (ii) SNPs located on the terminal region of the scaffolds and with less a than 75 bp flanking region, (iii) SNPs located within a distance of 100 bp to the target SNP, (iv) the insertion or deletion variant (InDel) and heterozygous SNPs, and (v) SNPs in the repetitive region. Among the SNPs amenable to Illumina Infinium assay development, SNPs were further selected based on functional roles (fatty acid biosynthesis, oil metabolism, disease resistance, and ricin biosynthesis) and genomic coverage (maximum possible representation of scaffolds with respect to the reference genome). The custom Infinium genotyping BeadChips assay was designed using Illumina Assay Design tools (ADT) for the selected SNPs. Finally, 6,000 SNPs that required only one bead type for genotyping and had assay design scores of >0.7 were selected for assay development.

### Validation of genotyping array

The technical performance of the array was evaluated by genotyping a panel of 314 inbred castor lines. The genomic DNA was extracted from 100 mg of leaf samples collected from plants of each of the inbred lines using the CTAB method. The DNA samples were quantified using Quibit and a NanoDrop-8000 UV-Vis Spectrophotometer (Thermo Fisher Scientific, USA). The samples’ quality was checked through agarose gel electrophoresis. Illumina Infinium genotyping assay was performed per standard procedures, which are briefly described below.

Genomic DNA samples from 314 lines in addition to two biological and two technical replicates, were amplified overnight. The amplified products were subsequently fragmented by a controlled enzymatic process. The DNA fragments were alcohol-precipitated and re-suspended. The BeadChips were prepared for hybridization in the capillary flow-through chamber. Samples were applied onto the BeadChips and incubated overnight. The DNA fragments of samples annealed to locus-specific oligomers during the hybridization step, wherein one bead type corresponded to a specific allele of the SNP locus. After hybridization, allelic specificity was conferred by enzymatic base extension. The products were subsequently fluorescently stained. The intensities of the beads’ fluorescence were detected by the iScan system (Illumina Inc., USA), and genotype calling was performed using GenomeStudio software (Illumina Inc., USA) with a GenCall score cut-off of 0.15 (as recommended by Illumina for Infinium data).

The genotyping array;s quality was assessed based on the call rate, reproducibility, and polymorphism detection ability of the SNPs in the array. Various genetic diversity parameters, namely, minor allele frequency (MAF), observed heterozygosity (Ho), gene diversity or expected heterozygosity (He), and polymorphic information content (PIC), were estimated using the software program PowerMarker version 3.25^41^.

### Construction of linkage map

The RILs of JC12 × 48-1 and DCS9 × RG1139, along with their parents, were genotyped using the SNP array developed in this study. Genotyping was done as described in section 2.5. The markers were assigned to linkage groups using Joinmap 3.0 software^42^ at the logarithm of the odds (LOD) threshold of >8. The ordering of loci within the linkage group was done using the software RECORD^43^. Map distances were calculated using the Haldane mapping function. The consensus map based on data from two populations was constructed using Mapfuser, a Shiny application at https://plantbreeding.shinyapps.io/mapfuser. Mapfuser constructs consensus genetic maps using LPmerge software^44^.

## Supplementary information

Supplementary Fig 1

Supplementary Table S1

Supplementary Table S2

Supplementary Table S3

Supplementary Table S4

## Data Availability

The raw sequence reads generated in this study are available at NCBI’s Sequence Read Archive (SRA) with BioProject ID: PRJNA513227 (http://www.ncbi.nlm.nih.gov/bioproject/513227) and accession Nos. SAMN10698912 to SAMN10698925.
