# Supplementary material for: Development and validation of an SNP genotyping array and construction of a high-density linkage map in castor
Source: Sci Rep. 2019 Feb 28;9:3003. doi: 10.1038/s41598-019-39967-9 (PMC6395776; doi:10.1038/s41598-019-39967-9)
Supplement: Supplementary file 1 — Supplementary Fig 1 [file 41598_2019_39967_MOESM1_ESM.pdf]

Development and validation of an SNP genotyping array and construction of a high-density linkage map in castor

S. Senthilvel*, Arpita Ghosh, Mobeen Shaik, Ranjan K. Shaw, Prashanth G. Bagali

S. Senthilvel, Mobeen Shaik, Ranjan K. Shaw
ICAR-Indian Institute of Oilseeds Research,
Rajendranagar, Hyderabad – 500030, India

Arpita Ghosh, Prashanth G. Bagali
Xcelris Labs Ltd., Xcellon building, Navrangpura,
Ahmedabad – 380009, India

*Corresponding author
S. Senthilvel
e mail: senthilvel.senapathy@icar.gov.in
Telephone: +91 40 24598116; Fax: +91 40 24017969

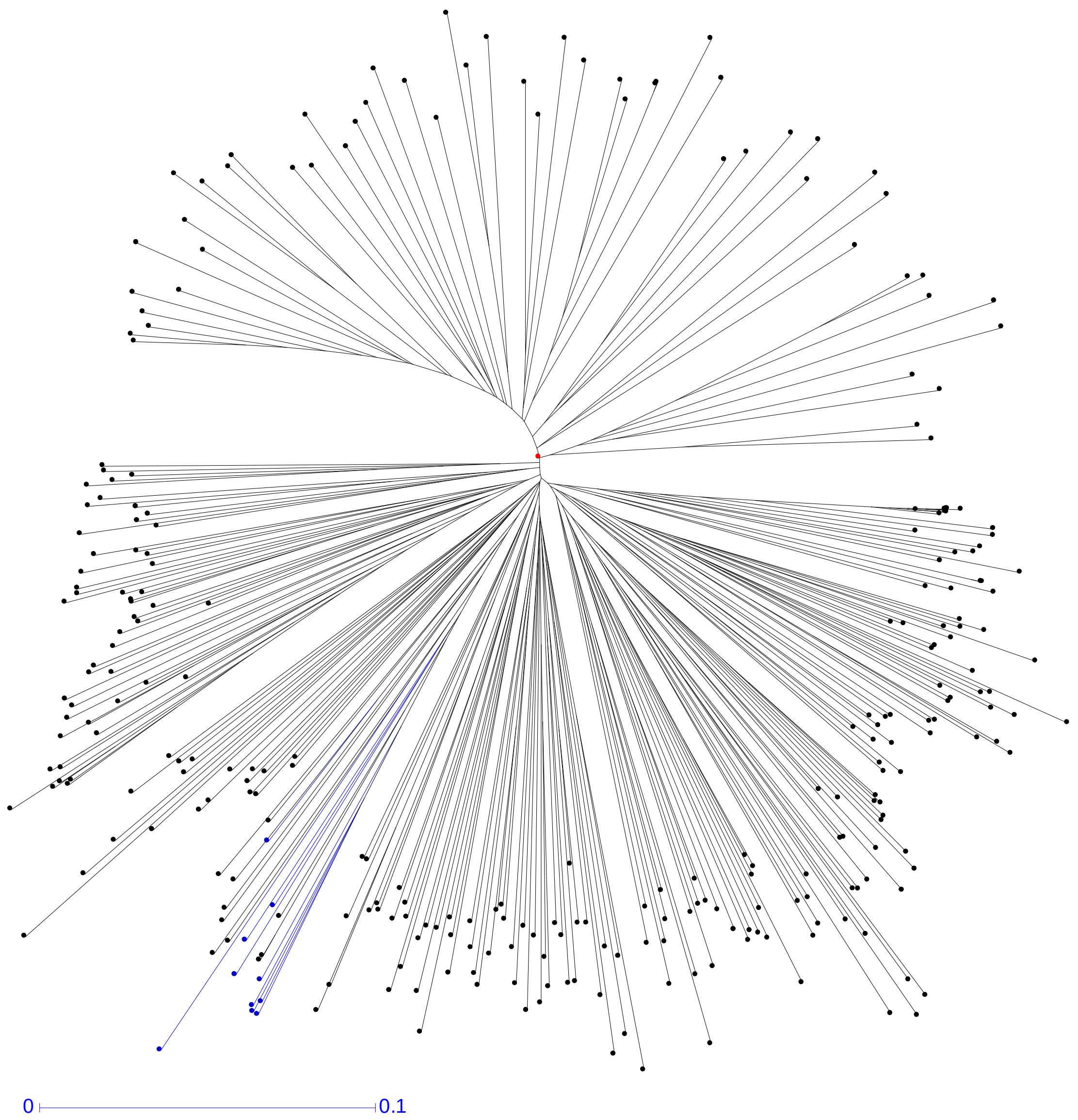

Supplementary Fig 1.Neighbour joining tree showing the genetic relationship of 314 castor inbred lines based on SNP genotypic data (DPC9 and its mutant selections are coloured in blue)
